# Significance of subcentimetre ^18^F-FDG PET/CT pulmonary abnormality in patients with known extrapulmonary malignancy

**DOI:** 10.2349/biij.6.4.e34

**Published:** 2010-10-01

**Authors:** AS Fathinul Fikri, WFE Lau

**Affiliations:** 1 Centre for Diagnostic Nuclear Imaging , Faculty of Medicine and Health Science, Universiti Putra Malaysia, Malaysia; 2 Department of Radiology, the University of Melbourne, and Centre for Molecular Imaging, the Peter MacCallum Cancer Centre, Australia

**Keywords:** Significance, Subcentimetre, Single Pulmonary Nodule, FDG-PET

## Abstract

The significance of a subcentimetre ^18^F-FDG PET/CT pulmonary abnormality in a patient with known extrapulmonary primary malignancy can have a major impact on the clinical management of the patient. The clinician’s reliance on the semi-quantitative and qualitative PET/CT analysis of the abnormality has, at times, led to untoward diagnostic problems, given the limited spatial resolution of PET for a small volume lesion performed as part of the standard PET/CT study. This paper highlights a case each of an FDG-positive and an FDG-negative focal pulmonary abnormality in a combined PET/CT study of patients with known extrapulmonary malignancy. © 2010 Biomedical Imaging and Intervention Journal. All rights reserved.

## INTRODUCTION

Subcentimetre pulmonary abnormality is not an uncommon incidental finding on Positron Emission Tomography/Computed Tomography (PET/CT) studies performed in patients with known extrapulmonary malignancy [1]. It may be difficult to ascertain the aetiology of such abnormalities on the PET/CT assessment and this may pose a diagnostic challenge to the physician when attempting to adopt an appropriate management plan. A subcentimetre pulmonary abnormality can have many aetiologies, ranging from benign lesions, such as granuloma or inflammatory lesions, to a malignant pathology, such as metastasis or primary lung malignancy [1]. ^18^F-Fluorodeoxyglucose (^18^F-FDG) PET has been used to triage the clinical significance of small pulmonary lesions, and helps to devise appropriate management strategies. However, some tumours with high malignant potential can have relatively low FDG-uptake [2]. Prediction of malignancy for small PET-negative nodule may be low and Joo et al. has reported that there is more than 19% risk of malignancy in subcentimetre FDG-negative nodules [1]. This is further substantiated by most reports, which have suggested that the reliability of minimal SUV value of a subcentimetre pulmonary abnormality is not compelling [3]. On the other hand, an FDG-positive pulmonary abnormality has a higher chance of being malignant. Using a semi-quantitative test in PET, the accuracy of standardised uptake value (SUV max), which has a cutoff of 2.5 to indicate a malignancy, improved with an increase in the diameter of the pulmonary nodule in question [2].

This paper presents one case each of a PET-positive and a PET-negative of a subcentimetre pulmonary abnormality in a combined PET/CT study and highlights the significance of their existence in patients with extrapulmonary malignancy.

## CASE REPORT 1

**Figure 1:**
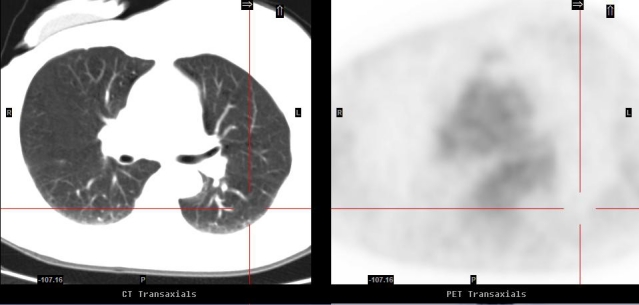
Correlative images of PET/CT (taken February 2007); The right PET image displayed an avid FDG (SUV max of 17.9) focus in the apical segment of the left lower lobe without any apparent nodules on the corresponding CT image on the left.

**Figure 2:**
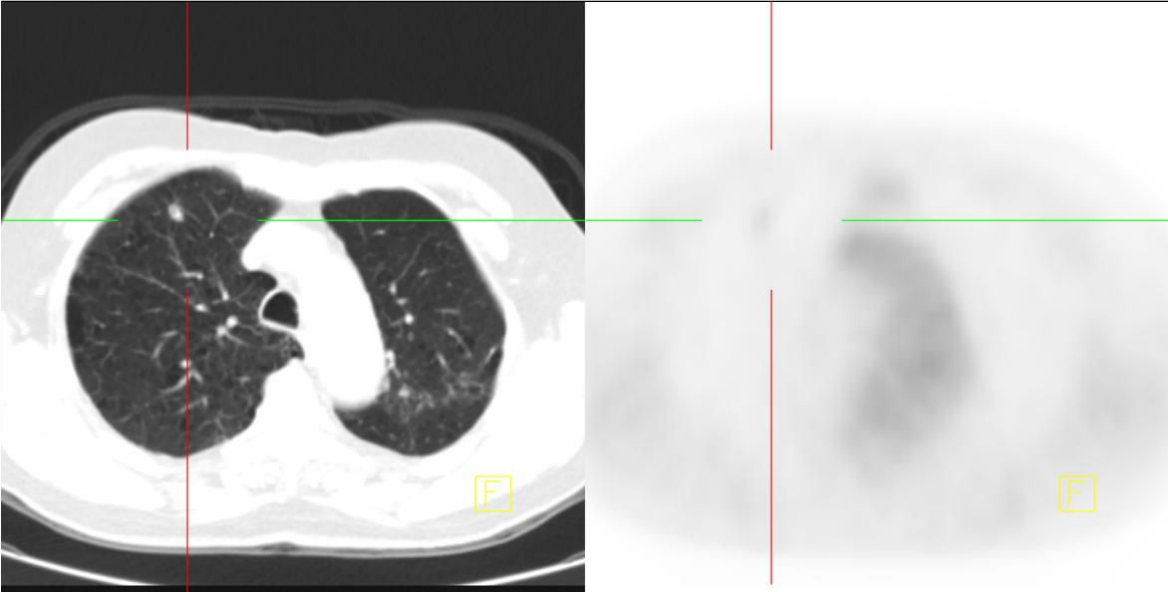
Correlative images of PET/CT (taken September 2007); The previously documented focal FDG uptake in the left lung has resolved, consistent with an FDG microembolus rather than metastatic disease.

A 61-year-old lady with a history of nodal positive right breast cancer was treated by surgery and chemotherapy in 1996. She was referred for a PET/CT in February 2007 with the query of locoregional recurrence because of recurrent chest wall pain. A PET/CT study revealed a highly intense focus in the apical segment of the left lower lobe on the PET images but there was no perceptible nodule on the corresponding CT images (Figure 1). Given the superior sensitivity of the co-registered CT images in the detection of pulmonary nodules, the PET abnormality was not considered to represent pulmonary metastasis and a provisional diagnosis of an FDG microembolus was made. The patient was managed conservatively. Follow-up PET/CT performed seven months later demonstrated complete resolution of the PET abnormality and reaffirmed the original diagnosis (Figure 2).

## CASE REPORT 2

**Figure 3:**
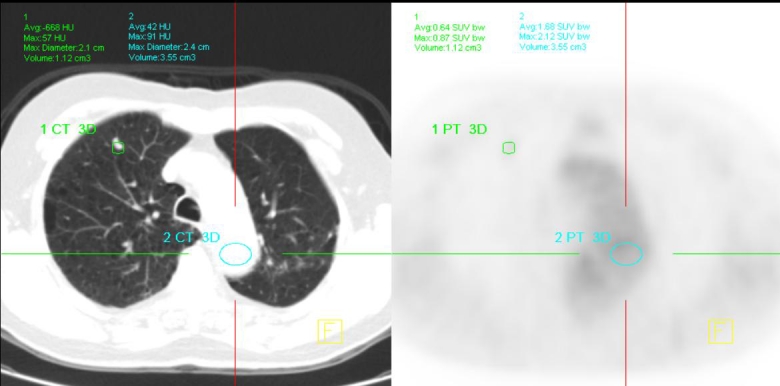
Correlative images of PET/CT revealed a 5 × 9 mm nodule in the anterior segment of the right upper lobe with minimal FDG avidity.

**Figure 4:**
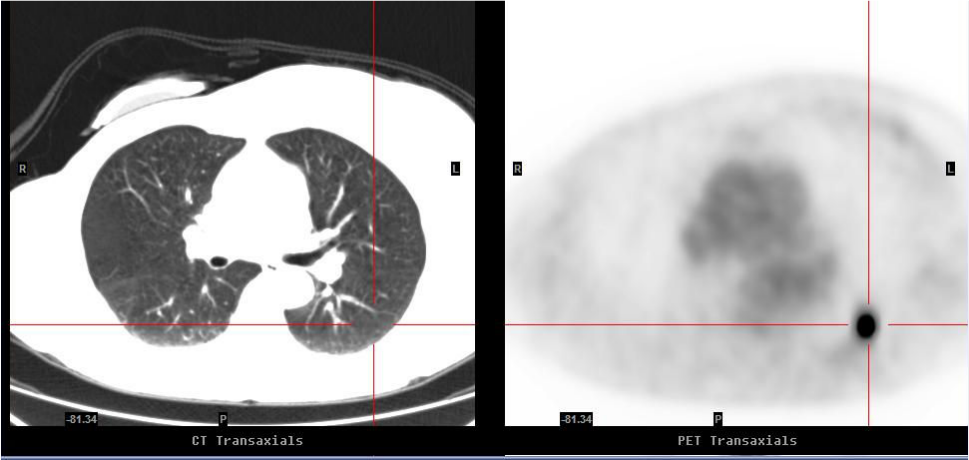
Correlative PET/CT images displayed that the SUV max of the right upper lobe nodule (0.9) is less than that of the mediastinal blood pool (2.1).

A 69-year-old male, with a history of Stage II colonic cancer resected two years prior, presented for a PET scan to assess a progressive right upper lobe (RUL) nodule on serial CTs. On PET, there was a 5 × 9 mm nodule with minimal FDG avidity (SUV max: 0.87), of which visual intensity is less than that of the mediastinal blood pool (Figures 3 and 4). The nodule was still considered to have a significant malignant potential given its small size and progressive nature on serial CTs despite its low FDG intrinsic nature. Biopsy of the lesion revealed a low grade adenocarcinoma, consistent with a primary lung cancer.

## DISCUSSION

The PET-positive SPN in Case Report 1 was considered to be a benign FDG microembolus given the absence of a nodule on the co-registered CT images, and this was confirmed by its spontaneous resolution on the follow up PET/CT study. An FDG-microembolus resulting from FDG-tracer contamination at the injection site is an uncommon phenomenon, which may be incidentally encountered during a routine PET/CT study [4]. This may be suspected when there are no corresponding nodules on the CT to account for a highly avid FDG focus, with the understanding that the co-registered CT images are invariably more sensitive than PET due to its superior contrast and spatial resolution in the lungs [2]. In general, a genuine FDG avid lung tumour must be seen on CT to be confirmed. Nonetheless, the presence of high FDG avidity in a pulmonary nodule seen on CT is highly predictive of malignancy; and a malignant final diagnosis is approximately 10 times more likely than a benign aetiology when PET results are rated ‘definitely malignant’ [5].

In the second case report, the subcentimetre pulmonary abnormality was considered a PET-negative lesion based on both semi-quantitative and qualitative criteria of its low FDG avidity when compared to the mediastinal blood pool [1, 5]. Its low intrinsic FDG avidity was, however, interpreted in the setting of a corresponding small progressive nodule on CT, suggesting a strong suspicion of a small volume or low grade malignancy [1, 5]. This is explained by the presence of relatively low metabolic activity in certain malignant nodules, as well as the effect of respiratory blurring and partial voluming in small nodules. These factors limit the ability of the co-registered PET/CT images to differentiate benign from malignant nodules [2]. The histological diagnosis of the nodule in question was a low grade adenocarcinoma which is known to exhibit low FDG uptake.

These two case reports illustrate how a reliable differentiation between malignant and benign nodules based on the co-registered PET/CT information is, at times, equivocal, and therefore serves as diagnostic challenges to the interpreter. Despite the high contrast resolution of the co-registered ^18^F-FDG PET in the detection of lung disease, its finite spatial resolution and the potential respiratory blurring limit a combined PET/CT ability to assess a subcentimetre nodule. In this context, the standard care of the combined PET interpretation with corresponding CT in evaluating a subcentimetre pulmonary nodule requires a meticulous correlation with the clinical course of the disease, the substantiation of the PET technology through the respiratory gating technique, and the use of alternative PET radiotracers, such as ^18^F-fluoro-thymidine (FLT).

## CONCLUSION

The significance of PET-positive and PET-negative subcentimetre pulmonary abnormality in a combined PET/CT study, at times, cause diagnostic uncertainty, which poses a problem in the disease management plan. These case reports document the difficulties of using the standard combined PET/CT to determine the malignancy potential of a subcentimetre pulmonary abnormality in patients with extrapulmonary malignancy.
